# The Feasibility of Enzyme Targeted Activation for Amino Acid/Dipeptide Monoester Prodrugs of Floxuridine; Cathepsin D as a Potential Targeted Enzym

**DOI:** 10.3390/molecules17043672

**Published:** 2012-03-26

**Authors:** Yasuhiro Tsume, Gordon L. Amidon

**Affiliations:** 1 Department of Pharmaceutical Science, University of Michigan, Ann Arbor, MI 48109, USA; Email: ytsume@umich.edu; 2 College of Pharmacy, The University of Michigan, 428 Church Street, Ann Arbor, MI 48109, USA

**Keywords:** prodrugs, Capan-2 cell, cathepsin, enzymatic activation, cell proliferation assay

## Abstract

The improvement of therapeutic efficacy for cancer agents has been a big challenge which includes the increase of tumor selectivity and the reduction of adverse effects at non-tumor sites. In order to achieve those goals, prodrug approaches have been extensively investigated. In this report, the potential activation enzymes for 5′-amino acid/dipeptide monoester floxuridine prodrugs in pancreatic cancer cells were selected and the feasibility of enzyme specific activation of prodrugs was evaluated. All prodrugs exhibited the range of 3.0–105.7 min of half life in Capan-2 cell homogenate with the presence and the absence of selective enzyme inhibitors. 5′-*O*-L--Phenylalanyl-L-tyrosyl-floxuridine exhibited longer half life only with the presence of pepstatin A. Human cathepsin B and D selectively hydrolized 5′-*O*-L-phenylalanyl-L-tyrosylfloxuridine and 5′-*O*-L-phenylalanyl-L-glycylfloxuridine compared to the other tested prodrugs. The wide range of growth inhibitory effect by floxuridine prodrugs in Capan-2 cells was observed due to the different affinities of prodrug promoieties to enyzmes. In conclusion, it is feasible to design prodrugs which are activated by specific enzymes. Cathepsin D might be a good candidate as a target enzyme for prodrug activation and 5′-*O*-L-phenylalanyl-L-tyrosylfloxuridine may be the best candidate among the tested floxuridine prodrugs.

## 1. Introduction

Prodrug applications have been utilized in cancer treatment for improving drug delivery, minimizing toxicity at non-target sites, maximizing therapeutic effect and increasing oral bioavailability [1,2,3]. Amino acid monoester prodrugs of antiviral agents like acyclovir and ganciclovir have been reported to improve their oral bioavailability by facilitating carrier-mediated transport [4,5,6,7]. Amino acid monoester prodrugs of the anti-cancer drug gemcitabine have also exhibited enhanced affinity to the oligopeptide transporter (PEPT1) [8]. The PEPT1 transporter has been extensively examined as a delivery site to improve oral drug absorption and the pro-moieties targeting this transporter have been investigated to improve their binding affinity [9,10,11].

The anticancer agent 5-fluoro-2′-deoxyuridine (floxuridine) has been clinically used in the treatment of colon carcinoma and colorectal cancer metastases to the liver. The mechanical action of 5-fluorouracil (5-FU) and floxuridine is well understood [12]. Floxuridine has a specific activity in DNA and has less cytotoxicity in RNA and, thus, this specificity of floxuridine minimizes adverse effects unlike 5-FU [13,14]. It has been reported that the potency and the growth inhibition of floxuridine is 10- to 100-fold better than those of 5-FU [15,16]. Hence, the protection of the glycosidic bond in floxuridine will produce better clinical outputs. Floxuridine, however, is rapidly converted to 5-FU by thymidine phosphorylase, which is ubiquitously observed in many tissues, including the liver [17]. Therefore, the protection of glycosidic bond of floxuridine leads to improve therapeutic efficiency. A variety of enzymes have been tested as the possible target enzyme to activate prodrugs because those prodrugs have to be activated in order to reveal their therapeutic effects. Irinotecan (CPT-11) is an anti-cancer prodrug of active metabolite SN-38 and has been approved for the treatment of colon cancer. CPT-11 has been investigated for specific activation of irinotecan at tumor sites by carboxylesterase to minimize toxicity in non-tumor sites [3,18,19,20,21]. The progression of technology can make it possible to examine the crystal structure of carboxylesterase and to design more efficient prodrugs activated by specific enzymes [18,22,23]. Highly expressed enzymes have been studied as possible enzyme targets in certain disease stages. The enzyme targeting for prodrug activation in various diseases has also been carried out. It has been reported that endopeptidase cathepsins have been upregulated in tumor cells and are expressed more than those in non-cancer cells [24,25,26]. Furthermore, the signaling pathways related to upregulation of cathepsins have been gradually recognized [27,28]. It is well documented that cathepsins B, D, and L are the most abundant proteases and the increased expression of those cathepsins plays a part in the invasion and metastasis in the tumor growth [29,30,31,32,33,34]. Therefore, cathepsin B, D, and L have a potential to be good targets for prodrug activation. Doxorubicin and daunorubicin prodrugs conjugated with peptides and amino acids show the less toxicity than their parent drugs, doxorubicin and daunorubicin, and are activated by hydrolytic enzymes like the cathepsins [35,36,37,38,39,40]. Capecitabine is a prodrug of 5-FU like floxuridine and is used for the treatment of breast and colorectal cancers. This prodrug, which requires a few activation steps by enzymes to show its therapeutic effect, has been investigated in its activation, distribution, and pharmacokinetics [41,42,43]. However, the avoidance of adverse effects in CPT-11 and capecitabine still has not been fully successful. The understanding of prodrug activation in tumor cells is necessary and leads to the design and production of more advanced prodrugs. Yet, the activation of prodrugs neither has been examined nor has been given adequate attention even though this is one of the most important steps in prodrug approaches.

In this report, the feasibility of cathepsin D targeting for the activation of amino acid/dipeptide monoester prodrugs of floxuridine and the capability of inhibiting cancer cell growth for those activated prodrugs are described. Also, those 5′-dipeptide monoester prodrugs of floxuridine are compared with the 5′-mono amino acid monoester prodrugs of floxuridine. The enzymatic activation of these prodrugs was evaluated to determine the effects of amino acid/dipeptide promoieties and esterification site for enzyme-mediated activation. The activation of translocated prodrugs in tumor cells, the enzymes involved in the prodrug activation, and the ability of tumor growth inhibition for tested prodrugs were discussed.

## 2. Results and Discussion

Prodrug approaches have been investigated to improve oral absorption and therapeutic effects of poorly permeant drugs [44]. Amino acid/dipeptide monoester prodrugs of antiviral and anticancer drugs such as gemcitabine, acyclovir, 2-bromo-5,6-dichloro-1-(β-D-ribofuranosyl)benzimidazole (BDCRB) and floxuridine have been synthesized and characterized in our previous reports [8,45,46,47,48,49,50,51]. The synthesis methods and characterization of those floxuridine prodrugs have been reported [49,50].

Lysosomal proteases called cathepsins may be attractive target enzymes because of high expression in tumor cells. It has been reported that lysosomal cathepsins, especially cathepsin B and D, are upregulated in tumors and redistributed to the outside of lysosomes such as cytosol and plasma membrane [28,52,53,54,55,56,57,58]. Thus, cathepsin B and D in tumors might be good target enzymes even though they are lysosomal enzymes. It has been reported that cathepsin D is present in all tissues but the expression levels significantly vary in the type of tissues and their physiological state [59]. The location and expression level of enzymes are important decisive factors to select possible target enzymes for prodrug activation. For this purpose, enzymes which are located in the plasma membrane and cytosol, as well as highly expressed enzymes in lysosomes, were selected as candidates of prodrug-activation enzymes. 

The identification and selection of the specific enzymes for prodrug activation along with designing prodrugs have been investigated in order to minimize adverse effects and improve therapeutic index [60,61,62,63,64]. One of the most common tools for performing high-throughput expression measurements would be a microarray analysis to select target candidates. The Affymetrix GeneChip (GeneChip Human Genome U133 Plus 2.0) was used to obtain the gene expression profile of top 50 enzymes in specific locations, cytosol and plasma membrane as well as highly expressed lysosomal enzymes, of Capan-2 cell. Cathepsin D, puromycin-sensitive aminopeptidase, TPP2, and DPP3 were chosen as candidates of target enzymes due to their locations and high expression levels of gene in Capan-2 cell (Table 1). The experiments concerning identifying activation enzymes for floxuridine prodrugs were performed at 37 °C in Capan-2 cell homogenates in pH 7.4 phosphate buffer with the presence and the absence of selective enzyme inhibitors.

**Table 1:** Expression levels of top 50 enzyme genes in plasma membrane and cytosol with cathepsins in pancreatic cancer cell line, capan-2 and AsPC-1, with Affymetrix GeneChip Human Genome U133 Plus 2.0.

Enzyme	Capan-2	AsPC-1
**Cathepsin B **	**1526.6**	38
Zinc Metalloproteinase	1057.9	48
Cathepsin H	913.4	58.6
**Cathepsin D**	**891.2**	3.7
gamma-Glutamyl Hydrolase	840.6	20.3
Calpastatin (CAST)	746.2	28.3
Aminopeptidase B	726.2	14.9
Leucine Aminopeptidase	700.7	15.1
Cathepsin C	593.4	17.6
**Puromycin Sensitive Aminopeptidase**	**530.2**	4.9
Prolylcarboxypeptidase	516.3	16.8
Aminoacylase 1	511.9	16.2
Cytochrome P450	432.4	13.5
*N*-Acylaminoacyl-Peptide Hydrolase	335.5	9.7
**Tripeptidyl peptidase II (TPP2)**	**296.7**	36.4
Cathepsin U	288.4	7.4
Aminopeptidase P	264	328.2
**Dipeptidylpeptidase III (DPP3)**	**257.5**	4.2
Leucine Aminopeptidase	238	20.7
Mitochondrial Intermediate Peptidase (MIPEP)	206.8	35
Aminopeptidase PILS (APPILS)	195.1	2.4
UDP-*N*-Acetylglucosamine-2-Epimerase (GNE)	180.4	42.3
Caspase 6	179	0.8
Sentrin-Specific Protease (SENP2)	176	116.5
Glycosylasparaginase	176	130.1
Carboxypeptidase D	168.2	57.3
Transmembrane Protease(TMPRSS4)	167.8	3.9
Cysteine Protease	167.1	17.1
Aspartyl Aminopeptidase (DNPEP)	163.6	24.7
Peptidase D (PEPD)	163.2	3.3
Heparan Sulfate (glucosamine) 3-O-Sulfotransferase 1 (HS3ST1)	157.1	5.3
Aspartylglucosaminidase	121.5	28.6
GPI Transamidase	119.6	112.6
SentrinSUMO-specific protease 3 (SENP3)	117.8	111.9
Caspase 3	114.8	5.1
Carboxy-Terminal Hydrolase	111.7	56
Caspase 8	108.8	49.8
Phosphatidylcholine 2-acylhydrolase (cPLA2)	108.3	3.3
Polypeptide 5 (RPS6KA5)	103.3	32.1
beta-Site APP-Cleaving Enzyme	100.2	178.3
Protease 5 (isopeptidase T) (USP5)	99.9	84
Arginyl Aminopeptidase (Aminopeptidase B)-Like 1 (RNPEPL1)	99.3	16.1
SentrinSUMO-Specific Protease (SENP1)	98.8	48.3
Carboxypeptidase M	97.4	7.8
Carboxypeptidase D	96.9	4.9
3-Phosphoinositide Dependent Protein Kinase-1 (PDPK1)	94.7	53.3
Putative Metalloglycoprotease	94	55.4
Aspartylglucosaminidase (AGA)	93.2	12.5
Carboxypeptidase 1	92.8	15
Matrix Metalloproteinase 14 (MMP14)	88.5	10.3

**BOLD:** selected for enzyme inhibition studies.

Table 2 displays the estimated half-lives (t_1/2_) obtained from the initial rates of hydrolysis by plotting the logarithm of remaining prodrugs as a function of time. All prodrugs showed shorter half-lives in cell homogenates than in pH 7.4 phosphate buffer suggesting enzyme catalyzed hydrolysis. No degradation of prodrugs was confirmed in lower than pH 6.0 phosphate buffer (data not shown). Amino acid monoester prodrugs of floxuridine and their parent drug were observed from 5′-*O*-L-valyl-L-phenylalanylfloxuridine, 5′-*O*-L-phenylalanyl-L-tyrosylfloxuridine and 5′-*O*-L-glycyl-L-leucyl-floxuridine. However, only floxuridine was observed from the rest of tested prodrugs. The stabilities of 5′-*O*-L-leucyl-L-glycylfloxuridine and 5′-*O*-L-valyl-L-phenylalanylfloxuridine were 1.4- to 8.3-fold and 0.8- to 1.7- fold better with the presence of enzyme inhibitors, respectively. However, significant differences among them could not be observed. Only 5′-*O*-L-glycyl-L-leucylfloxuridine exhibited no change in its stability in all conditions. This result is indicating that there are many enzymes to activate 5′-*O*-L-glycyl-L-leucylfloxuridine or/and selected enzyme inhibitors did not effectively inhibit enzymatic activation of this prodrug. 5′-*O*-L-phenylalanyl-L-glycylfloxuridine and 5′-*O*-L-phenyl-alanylfloxuridine exhibited 2.4- to 7.0-fold and 5.1- to 20.2-fold longer half lives with the presence of enzyme inhibitors. This suggests that those targeted enzymes associate to those prodrug activations. However, activation enzymes for those two prodrugs could not be specified. The half life of 5′-*O*-L-phenylalanyl-L-tyrosylfloxuridine in Capan-2 cell homogenate with the presence of pepstatin A was significantly prolonged, while the half lives of this prodrug with other enzyme inhibitors were not improved. Thus, the activation enzyme of 5′-*O*-L-phenylalanyl-L-tyrosylfloxuridine would be specific and cathepsin D might be the one of major activation enzymes for this prodrug activation.

**Figure 1:**
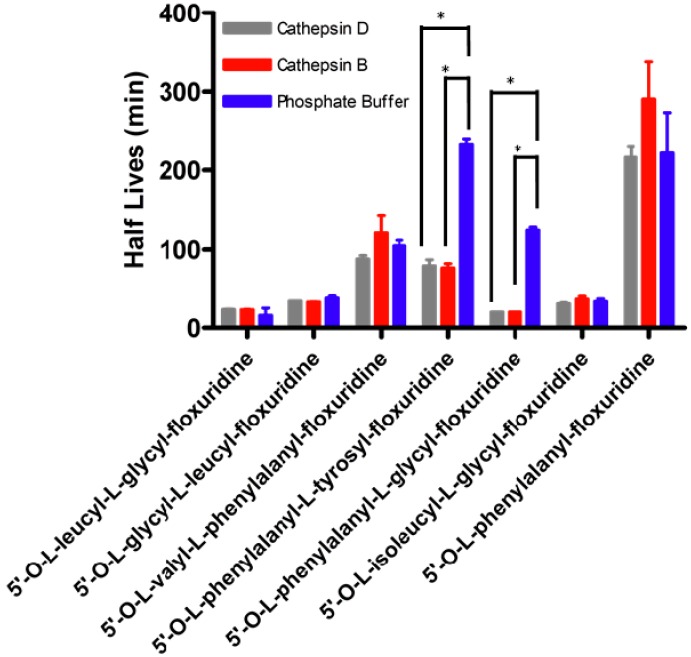
Prodrug stability in human cathepsin B and D. Values presented are the mean ± s.d., n = 4, (*p < 0.01; *t*-test).

The high expression levels of cathepsins in tumors have been reported [65,66,67,68]. It has been investigated if the protein levels of cathepsin B and D would associate with pathological parameters in order to assess the difference in protein levels of cathepsins as possible tumor biomarkers [34,69,70,71]. Cathepsin B, one of the highest gene expressions in enzymes of Capan-2 cell, is a cysteine endopeptidase and reportedly has similar activity to carboxypeptidase and peptidyl-dipeptidase [72,73,74]. On the other hand, cathepsin D has been reported that it preferentially hydrolyzes the peptide bond of bulky hydrophobic amino acids and the peptide bond between Phe-Phe of Bz-Arg-Gly-Phe-Phe-Pro-4MbNA [75,76,77]. The metabolic stability of floxuridine prodrugs was assessed using human cathepsin B and D in pH 7.4 phosphate buffer. Figure 1 shows the stability of floxuridine prodrugs against cathepsin B and D compared with the absence of those endopeptidases, which representing chemical stability of prodrugs. All tested floxuridine prodrugs with exception of 5′-*O*-L-phenylalanyl-L-glycyl-floxuridine and 5′-*O*-L-phenylalanyl-L-tyrosylfloxuridine did not exhibit differences in their half lives. Only 5′-*O*-L-phenylalanyl-L-glycylfloxuridine and 5′-*O*-L-phenylalanyl-L-tyrosylfloxuridine showed significant degradation by cathepsin B and D. The production of amino acid mono ester prodrug of floxuridine was clearly observed from 5′-*O*-L-phenylalanyl-L-tyrosyl-floxuridine. However, the enzyme specificity between cathepsin B and D in prodrug activation for those two prodrugs was not observed. As Offermann *et al.* have reported, aromatic amino acid incorporated prodrugs would be possible substrates for cathepsin B and D but the result showed that 5′-*O*-L-valyl-L-phenylalanyl-floxuridine was not a good substrate for cathepsins despite the incorporation of phenylalanine in the dipeptide promoiety [76]. The differences in the half lives of amino acid monoester prodrug of floxuridine, 5′-*O*-L-phenylalanylfloxuridine, was not observed with cathepsin enzymes. Thus, this result indicates that not only N-terminus aromatic amino acid but also dipeptide are required to be a substrate for cathepsins.

**Table 2:** Prodrug stability in capan-2 cell homogenate with the presence and the absence of inhibitors.

	Half Life (min)					
Prodrug	Phosphate Buffer pH 7.4	Capan-2 Cell Homogenate	Cathepsin D Inhibitor	Aminopeptidase Inhibitor	TPP 2 Inhibitor	DPP 3 Inhibitor
5'-*O*-L-leucyl-L-glycylfloxuridine	23.1 ± 4.1 ^§^	3.9 ± 1.1	7.9 ± 0.7	14.0 ± 4.3	5.6 ± 0.6	32.2 ± 9.3
5'-*O*-L-glycyl-L-leucylfloxuridine	35.7 ± 0.9 ^§^	29.2 ± 0.7	32.2 ± 0.1	33.0 ± 0.4	21.0 ± 0.9	26.5 ± 17.5
5'-*O*-L-valyl-L-phenylalanyl-floxuridine	104.7 ± 7.0 ^§^	56.2 ±12.8	76.4 ±6.1	93.6 ± 29.3	46.1 ± 1.9	67.7 ± 15.1
5'-*O*-L-phenylalanyl-L-tyrosyl-floxuridine	233.9 ± 6.6 ^§^	42.8 ± 0.0	105.7 ± 12.8 *	54.7 ± 2.8	42.2 ± 1.3	47.2 ± 3.6
5'-*O*-L-phenylalanyl-L-glycyl-floxuridine	132.1 ± 10.2 ^§^	4.3 ± 0.9	10.2 ± 3.0	25.7 ± 0.4 *	19.6 ± 0.4 *	30.1 ± 10.0
5'-*O*-L-phenylalanylfloxuridine	187.0 ± 19.0 ^§^	3.0 ± 0.1	60.6 ± 4.6*	48.7 ± 0.37 *	15.3 ± 0.6 *	28.9 ± 10.7

* <0.01; cathepsin D inhibitor–pepstatin A, aminopeptidase inhibitor–puromycin, TPP2 (tripeptidylpepptidase 2) inhibitor-H-Ala-Ala-Phe-CMK CF_3_CO_2_H, DPP3 (dipeptidylpeptidase 3)–1,10-phenanthroline. Asterisks indicate significant difference in half lives in capan-2 cell homogenate with an inhibitor to ones without inhibitors. Values presented are the mean ± s.d. (*p < 0.01; *t*-test). ^§^ from Reference [50].

**Figure 2:**
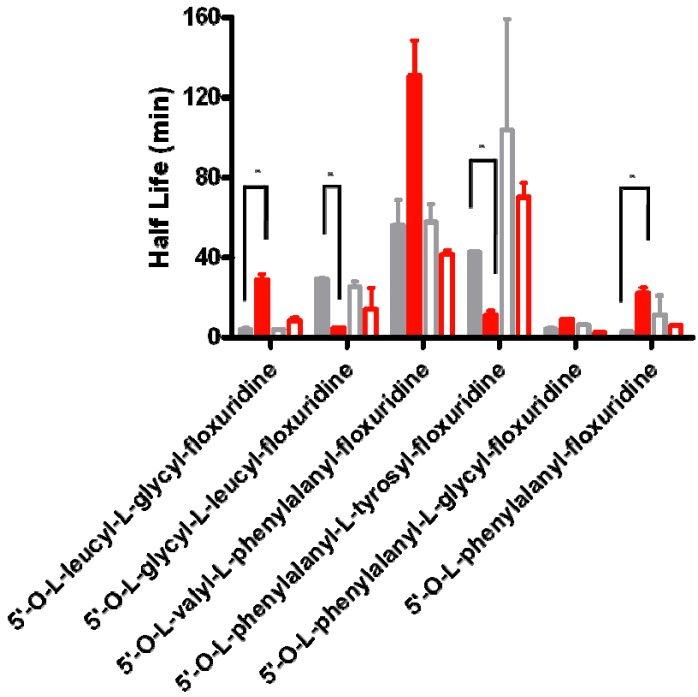
The difference of prodrug stability in the cytosolic fraction of cell homogenate and in whole cell homogenate of Capan-2 and Caco-2 cells. Gray bars represent the prodrug stability in whole cell homogenate and red bars represent the prodrug stability in the cytosolic fraction of cell homogenate. Closed bars represent the prodrug stability in Capan-2 cells and open bars represent the prodrug stability in Caco-2 cells. Values presented are the mean ± s.d., n = 4, (* *p* < 0.01; *t*-test).

In stability studies of test compounds in cell homogenates, the location of enzymes in living cells is not considered and the inaccessible enzymes *in vivo* condition may be able to degrade those analytes in this *in vitro* setting. Therefore, the result of prodrug stabilities in cell homogenate may be different to one *in vivo*, where all enzymes are arranged in all specific locations. The experiments of prodrug activation concerning the location of enzymes in cells were performed at 37 °C in Capan-2 cell homogenates and in cytosolic enzyme extracts. For the comparison purpose, the same experiments were performed at 37 °C in Caco-2 cell homogenates and in cytosolic enzyme extracts. All tested prodrugs except 5′-*O*-L-phenylalanyl-L-tyrosylfloxuridine and 5′-*O*-L-glycyl-L-leucylfloxuridine showed 2.0- to 7.4-fold longer half lives in cytosolic extract than ones in Capan-2 cell homogenate in pH 7.4 phosphate buffer (Figure 2). On the other hand, all tested prodrugs except 5′-*O*-L-leucyl-L-glycylfloxuridine exhibited shorter half lives in cytosolic extract than ones in Caco-2 cell homogenate in pH 7.4 phosphate buffer. All tested prodrugs in Caco-2 cells did not exhibit significant difference between in whole cell homogenate and in the cytosolic extract. The difference between them was minimal (1.4- to 2.5 fold). Floxuridine and amino acid mono ester prodrugs of floxuridine were observed from 5′-*O*-L-valyl-L-phenylalanylfloxuridine, 5′-*O*-L-phenylalanyl-L-tyrosylfloxuridine and 5′-*O*-L-glycyl-L-leucylfloxuridine but only floxuridine was detected from 5′-*O*-L-leucyl-L-glycyl-floxuridine, 5′-*O*-L-isoleucyl-L-glycylfloxuridine, 5′-*O*-L-phenylalanyl-L-glycylfloxuridine, and 5′-*O*-L-phenylalanylfloxuridine. In Capan-2 cells, the stabilities of 5′-*O*-L-phenylalanyl-L-glycylfloxuridine and 5′-*O*-L-phenylalanylfloxuridine were improved 7.4- and 7.3-fold, while the ones of 5′-*O*-L- phenylalanyl-L-tyrosylfloxuridine and 5′-*O*-L-glycyl-L-leucylfloxuridine were declined 3.9- and 6.0-fold, respectively. The latter result suggests: (1) there is the same amount of protein but there is difference in the population of enzymes between cell homogenate and cytosolic fraction; (2) there are more enzymes in cytosol extract to activate 5′-*O*-L-phenylalanyl-L-tyrosylfloxuridine and 5′-*O*-L-glycyl-L-leucylfloxuridine than ones to activate other prodrugs; and (3) the redistribution/trafficking of upregulated lysosomal enzymes to outside of lysosomal compartments [53,54,55,78]. Those results indicate that the prodrug stabilities *in vitro* might mislead prodrug stabilities *in vivo* due to the difference in cellular enzymatic population and location.

**Figure 3:**
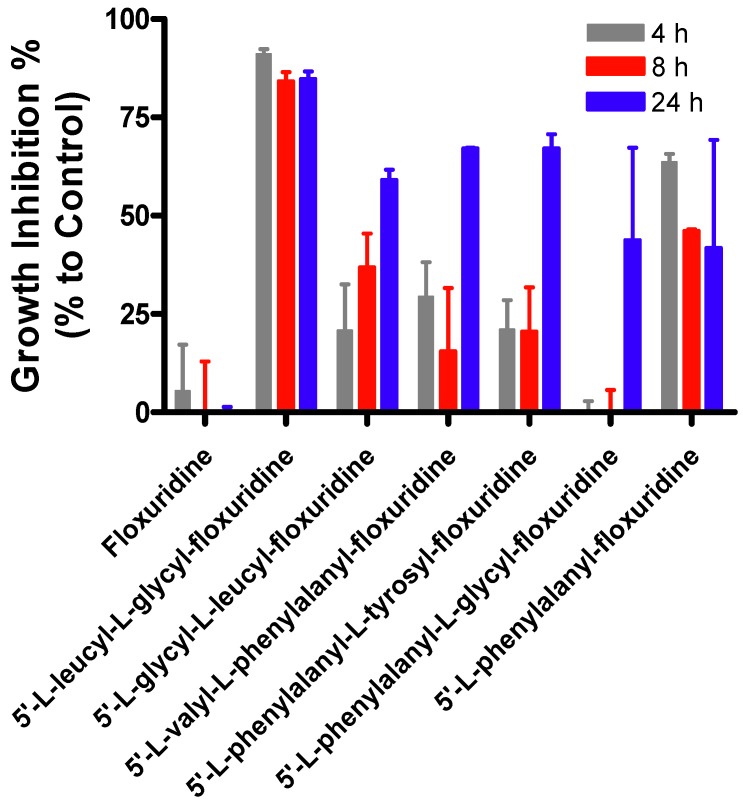
The growth inhibitory effects (%) of floxuridine prodrugs with varied post exposure time (4, 8, and 24 h). Values presented are the mean ± s.d. (n = 4).

The percentage of growth inhibition for amino acid/dipeptide monoester prodrugs of floxuridine in Capan-2 cell was evaluated via 2,3-bis[2-methoxy-4-nitro-5-sulfophenyl]-2*H*-tetrazolium-5-carboxanilideinner salt (XTT) assays (Figure 3). The growth inhibitory effect of tested floxuridine prodrugs after 4 h drug treatment range from 0.0–91.1% at 4 h, 0.0–84.2% at 8 h, and 41.8–84.8% at 24 h in Capan-2 cell lines. 5′-*O*-L-leucyl-L-glycylfloxuridine and 5′-*O*-L-phenylalanylfloxuridine immediately inhibited the growth of tumor cells while the rest of floxuridine prodrugs needed a certain time to be activated for their inhibitory effects. While specific activation enzymes for 5′-*O*-L-leucyl-L-glycylfloxuridine and 5′-*O*-L-phenylalanylfloxuridine have not been identified, those enzymes may be highly localized at the plasma membrane and cytosol. Thus, the activation of those prodrugs took place immediately after translocation of prodrugs into the cells and started inhibiting tumor growth. Leucine and phenylalanine are reportedly good substrates for aminopeptidases and carboxylesterase, respectively.

Thus, 5′-*O*-L-leucyl-L-glycylfloxuridine and 5′-*O*-L-phenylalanylfloxuridine might be activated by aminopeptidases and carboxylesterases, which are highly expressed at the plasma membrane and cytosol, to inhibit the growth of cancer cells [79,80]. 5′-*O*-L-phenylalanyl-L-glycyl-floxuridine, which exhibited a shorter half life in cell homogenate, did not exhibit the growth inhibition of cancer cells at 4 h and 8 h, suggesting that 5′-*O*-L-phenylalanyl-L-glycylfloxuridine might be more stable *in vivo* cell condition than one in cell homogenate. Those results imply that floxuridine prodrugs have to be activated in order to inhibit tumor growth. The prodrug activation would be initiated at different time periods due to the affinity between promoieties and enzymes and the location and population of prodrug-activation-enzymes.

The sites of prodrug activation in cells, the duration for activating prodrugs, the enzyme population, and the activation enzymes based on promoieties are all different. Therefore, *in vitro* experiments have to be carefully designed and prodrug approaches should be monitored by not only the amount of delivered prodrug but also prodrug activation and their therapeutic effect.

## 3. Experimental

### 3.1. Materials

Floxuridine was obtained from Lancaster (Windham, NH, USA). The *tert*-butyloxycarbonyl (Boc) protected amino acids Boc-L-phenylalanine, Boc-L-glycyl-leucine, Boc-L-phenylalanyl-glycine, Boc-L-leucyl-glycine, Boc-L-isolucylglycine, Boc-L-valyl-phenylalanine, and Boc-L-phenylalanyl-tyrosine were obtained from Chem-Impex (Wood Dale, IL, USA). Pepstatin A, cathepsin B (human liver), and cathepsin D (human liver) were obtained from Sigma Chemical Co (St. Louis, MO, USA). Enzyme inhibitors were purchased from EMD Chemicals (Gibbstown, NJ, USA). High-performance liquid chromatography (HPLC) grade acetonitrile was obtained from Fisher Scientific (St. Louis, MO, USA). *N,N*-dicyclohexylcarbodiimide, *N,N*-dimethylaminopyridine, trifluoroacetic acid (TFA), and all other reagents and solvents were purchased from Aldrich Chemical Co. (Milwaukee, WI, USA). Cell culture reagents were obtained from Invitrogen (Carlsbad, CA, USA) and cell culture supplies were obtained from Corning (Corning, NY, USA) and Falcon (Lincoln Park, NJ, USA). The Affymetrix GeneChip Human Genome U133 Plus 2.0 was purchased from Affymetrix Inc. (Santa Clara, CA, USA). All chemicals were either analytic or HPLC grade.

### 3.2. Floxuridine Prodrug Synthesis

The synthesis and characterization of 5′-monoamino acid and dipeptide ester prodrugs of floxuridine have been reported previously [46,49,50]. Briefly, Boc-protected amino acid and dipeptide (1,1 mmol), *N,N*-dicyclohexylcarbodiimide (1,1 mmol), and *N,N*-dimethylaminopyridine (0.1 mmol) were allowed to react with floxuridine (1 mmol) in dry DMF (7 mL) for 24 h. The reaction progress was monitored by TLC (ethyl acetate as eluent). The reaction mixture was filtered and DMF was removed under vacuum at 40 °C. The residue was extracted with ethyl acetate (30 mL) and washed with water (2 × 20 mL), and saturated NaCl (20 mL). The organic layer was dried over MgSO_4_ and concentrated under vacuum. The reaction yielded a mixture of 3′-monoester, 5′-monoester, and 3′,5′-diester floxuridine prodrugs. The three spots observed on TLC were separated and purified using column chromatography (dichloromethane/methanol, 20:1). Fractions from each spot were concentrated under vacuum separately. The Boc group was cleaved by treating the residues with TFA-dichloromethane (1:1, 5 mL). After 4 h, the solvent was removed and the residues werereconstituted with water and lyophilized. The TFA salts of amino acid/dipeptide monoester prodrugs of floxuridine were obtained as white fluffy solids. The combined yield of floxuridine prodrugs was ~60%. HPLC was used to evaluate the prodrug purity. Prodrugs were between 90% and 99% pure. These prodrugs were easily separated from parent drug by HPLC. Electrospray ionization mass spectra(ESI-MS) were obtained on a Thermoquest LCQ ESI-MS. The observed molecular weights of all prodrugs were found to be consistent with that required by their structure. The structural identity of the prodrugs was then confirmed using proton nuclear magnetic resonance spectra (^1^H-NMR). ^1^H-NMR spectra were obtained on a 300 MHz Bruker DPX-300 NMR spectrometer.

### 3.3. Cell Culture

Capan-2 cells (passages 50–58) from American Type Culture Collection (Rockville, MD) were routinely maintained in RPMI-1640 containing 10% fetal bovine serum. Caco-2 cells (passages 30–33) from American Type Culture Collection (Rockville, MD, USA) were routinely maintained in DMEM containing 10% fetal bovine serum, 1% nonessential amino acids, 1 mmol/L sodium pyruvate, and 1% L-glutamine. Cells were grown in an atmosphere of 5% CO_2_ and 90% relative humidity at 37 °C.

### 3.4. Affymetrix Oligonucleotide Array

Capan-2 cells were cultured at 37 °C, 5% CO_2_ in RPMI-1640 supplemented with 10% fetal bovine serum. Our group previously described the preparation of Capan-2 total RNA samples [81,82]. The gene expression profile of Capan-2 cell was obtained using Affymetrix GeneChip Human Genome U133 Plus 2.0.

*Hydrolysis StudiesPreparation of Capan-2 cell homogenates*. Preparation of cell homogenates has been reported previously [49]. Confluent Capan-2 cells (2 plates; 150 mm × 25 mm plate) were rinsed with saline twice. The cells were washed off with 5 mL of pH 7.4 phosphate buffer (10 mmol/L) and the lysed cells by ultrasonication (Micro ultrasonic cell disrupter Model KT40, Kontes, Vineland, NJ, USA) were spun down by centrifugation.

*Preparation of Cytosolic Enzyme Extract. *Confluent Caco-2 cells and Capan-2 cells (2 plates; 150 mm × 25 mm plate) were rinsed with saline twice. The cells were washed off with 1 mL of pH 7.4 phosphate buffer (10 mmol/L) and the enzymes were extracted through 30 gauge needle 10 times in ice-cold bath. Cell suspension was spun at 10,000 × *g* for 2 h at 4 °C and the supernatant was spun at 39,000 × *g* for 2 h at 4 °C. Protein amount was quantified with the Bio-Rad (Hercules, CA, USA) DC Protein Assay using bovine serum albumin as a standard. The protein amount was adjusted to 500 µg/mL and the hydrolysis reactions were carried out in 96-well plates (Corning). Caco-2 cells and Capan-2 cell suspensions (250 µL) were placed in triplicate wells either presence or absence of various inhibitors, the reactions were started with the addition of substrate (200 µM), and wells were incubated at 37 °C for 120 min (time points; 0, 5, 10, 30, 60, and 120 min). At each time point, aliquot sample (35 µL) was removed and added to 150 µL of acetonitrile (ACN) with 0.1% TFA to prevent the further degradation. The mixtures were filtered with a 0.45 µm filters at 1,000 × *g* for 10 min at 4 °C. The filtrate wasthen analyzed via reverse-phaseHPLC.

*Prodrug stability with Cathepsin B and D*. Lyophilized enzyme powder was dissolved and adjusted to 2.5 mg/mL of pure enzyme, human cathepsin B and D. The hydrolysis reactions were immediately started by adding substrates (200 µM) in 96-well plates (Corning). The reactions were carried out at 37 °C for 120 min (time points; 0, 5, 10, 30, 60, and 120 min). At each time point, aliquot sample (35 µL) was removed and added to 150 µL of acetonitrile (ACN) with 0.1% TFA. The mixtures were filtered with a 0.45 µm filters at 1,000 × *g* for 10 min at 4 °C. The filtrate was then analyzed via reverse-phase HPLC.

### 3.5. Data Analysis

The initial rates of hydrolysis were used to obtain the apparent first-order rate constants and estimate the half-lives. The apparent first-order degradation rate constants of various floxuridine prodrugs at 37 °C were determined by plotting the logarithm of prodrug remaining as a function of time. The slopes of these plots are related to the rate constant, k, and given by:

k = 2.303 × slope (log C *vs.* time) (1)

The degradation half-lives were then estimated by the equation:

t_1/2_ = 0.693/k (2)

Statistical significance was evaluated with GraphPad Prism version 3.0 by performing one-way analysis of variance with post-hoc Tukey’s test to compare means. A *p* value of <0.01 was considered significant.

### 3.6. HPLC Analysis

The concentrations of prodrugs and their metabolites were determined on a Waters HPLC system (Waters, Inc., Milford, MA, USA). The HPLC system consisted of two Waters pumps (model 515), a Waters autosampler (WISP model 712), and a Waters UV detector (996 photodiode array detector). The system was controlled by Waters Millennium 32 software (version 3.0.1). Samples were injected onto a Waters Xterra C_18_ reverse-phase column (5 µm, 4.6 × 250 mm) equipped with a guard column. The compounds were eluted using gradient method. All prodrugs were run with the solvent B gradient changing 0% to 56% at a rate of 2%/min during a 28-min run. Standard curves generated for each prodrug and their parent drugs were used for quantitation of integrated area under peaks.

### 3.7. Cell Proliferation Assays

Cell proliferation studies were conducted with Capan-2 cell lines. The cells were seeded into 96-well plates at 125,000 cells per well and allowed to attach/grow for 24 h before drug solutions were added. The culture medium (RPMI-1640 + 10% fetal bovine serum) was removed and the cells were gently washed once with sterile pH 6.0 uptake buffer (145 mM NaCl, 0.5 mM MgCl_2_, 1 mM NaH_2_PO_4_, 1 mM CaCl_2_, 3 mM KCl, 5 mM glucose, and 5 mM MES). Floxuridine prodrugs and floxuridine were diluted in pH 6.0 uptake buffer to 4 mmol/L using no drug as a 100% viability control. The wash buffer was removed and 25 µL drug solution per well were added and incubated at 37 °C for 4 h in the cell incubator. After this time, the drug solutions were removed and the cells were again gently washed twice with sterile uptake buffer. RPMI-1640 was then added to each well after washing. The cells were allowed to recover for 4 h, 8 h, or 24 h before evaluating cell viability via 2,3-bis[2-methoxy-4-nitro-5-sulfophenyl]-2*H*-tetrazolium-5-carboxanilide inner salt (XTT) assays. A mixture (30 µL) containing 2,3-bis[2-methoxy-4-nitro-5-sulfophenyl]-2*H*-tetrazolium-5-carboxanilide inner salt in sterile RPMI-1640 without phenol red (1 mg/mL) and phenazine methosulfate (*N*-methyldibenzopyrazine methyl sulfate) (0.383 mg/mL) reagents were added to the cells and incubated at 37 °C for 1 h for the color to develop. Absorbance readings at 450 nm were recorded.

## 4. Conclusions

In summary, it is feasible to design prodrugs which are activated by specific enzymes. The prodrug stabilities in cell homogenate do not necessary reflect their stabilities *in vivo*. Cathepsin D might be a good candidate as a target enzyme for prodrug activation because of its upregulation and redistribution to other cellular compartments in tumor cells and its substrate specificity. The results of stability studies with the presence of enzyme inhibitors indicate that there are particular enzymes activating 5′-*O*-L-phenylalanyl-L-tyrosyl-floxuridine and 5′-*O*-L-phenylalanyl-L-glycylfloxuridine. Cathepsin B and D significantly activated 5′-*O*-L-phenylalanyl-L-tyrosyfloxuridine and 5′-*O*-L-phenylalanyl-L-glycyl-floxuridine to produce floxuridine. Our studies demonstrate that cathepsin D can significantly contribute to the activation of 5′-*O*-L-phenylalanyl-L-tyrosylfloxuridine. For tumors that express large amounts of cathepsins, it is likely that a substantial proportion of 5′-*O*-L-phenylalanyl-L-tyrosyl-floxuridine is hydrolyzed by cathepsin D. Taken together, our experimental results demonstrate that cathepsin D has the significant ability to activate dipeptide monoester prodrug of floxuridine, 5′-*O*-L-phenylalanyl-L-tyrosylfloxuridine, and, therefore, has the potential to be a target enzyme for prodrug activation in tumors. 5′-*O*-L-phenylalanyl-L-tyrosylfloxuridine has the capability of being a cathepsin D-targeted prodrug for enzymatic activation and exhibits the respectable growth inhibition of cancer cells. In conclusion, with the consideration of chemical/enzymatic stability of those prodrugs, 5′-*O*-L-phenylalanyl-L-tyrosylfloxuridine may be the best candidate for *in vivo* tumor reduction studies because this prodrug would be stable enough to stay longer in the systemic circulation to reach target sites for the activation by the specific enzymes. With careful evaluations, the prodrug approaches designed to ameliorate the toxicity of anti-cancer drugs and to maximize prodrug activation based on enzyme specificity are feasible.
